# The Relation of Diabetes Type 2 with Sexual Function among Reproductive Age Women in Iran, a Case-Control Study

**DOI:** 10.1155/2017/4838923

**Published:** 2017-04-06

**Authors:** Poorandokht Afshari, Shiva Yazdizadeh, Parvin Abedi, Homayra Rashidi

**Affiliations:** ^1^Midwifery Department, Reproductive Health Promotion Research Center, Ahvaz Jundishapur University of Medical Sciences, Ahvaz, Iran; ^2^Endocrinology Department, Diabetes Research Center, Ahvaz Jundishapur University of Medical Sciences, Ahvaz, Iran

## Abstract

*Background.* Diabetic patients are at the greater risk of retinopathy, nephropathy, neuropathy, and sexual dysfunction compared to the general population.* Objective.* The aim of this study was to evaluate the sexual dysfunction in type 2 diabetes reproductive age women in Iran.* Method.* This was a case-control study carried out on 130 women with type 2 diabetes and 130 healthy women. The type 2 diabetes diagnosis was confirmed with abnormal fasting blood sugar, abnormal random blood sugar test, and abnormal level of HbA1C. Eligible women were requested to complete a demographic questionnaire and female sexual function index (FSFI). The chi-square test, independent *t*-test, and Multivariate Analysis of Covariance (MANCOVA) were used for analyzing data.* Results.* Results of this study showed that diabetic women had significantly lower sexual desire, arousal, lubrication, and orgasm and more pain compared to the healthy women (*p* < 0.05). Also diabetic women had lower sexual satisfaction compared to the healthy women (*p* = 0.002). The total score of sexual function was significantly lower in the diabetic women compared to the healthy women (21.25 ± 7.04 versus 22.43 ± 7.6, *p* = 0.004).* Conclusion.* Results of this study showed that the score of all dimensions of sexual function in diabetic patients was lower than that in healthy women. Education and counseling about controlling diabetes and sexual function among diabetic women in reproductive age are recommended.

## 1. Introduction

Type 2 diabetes is a chronic disease with an adult-onset and mostly is independent of insulin [1]. Type 2 diabetes may happen in condition that the body resisted the effects of insulin or there is not enough secretion of insulin [2]. Globally, 285 million people are affected with type 2 diabetes, which is anticipated to reach 438 million by the year of 2030 [3]. The prevalence of diabetes in Asian countries due to the rapid development and urbanization is expected to be nearly 60% of the world's diabetic population [4]. The prevalence of type 2 diabetes in Iranian population >40 years has been reported as 24% where the risk in women is greater than men (*p* < 0.001) [5]. In a study on 14,993 people in Yazd province, Iran, results showed that the prevalence of type 2 diabetes was 16.3% and age and sex were important related factors [6].

Type 2 diabetes may have complications such as microvascular damage, with the highest incidence for retinopathy [7], nephropathy [8], and neuropathy [9]. Due to the damage to nerves and blood vessels in uncontrolled diabetes, sexual dysfunction in men and women may happen [10]. A study was conducted by Enzlin et al. on 120 women with type 1 diabetes and 180 healthy women to evaluate the relation of sexual dysfunction with type 1 diabetes. Results showed that women with diabetes were more likely to have sexual dysfunction (27% versus 15%, *p* = 0.04) and only lubrication showed significant difference between two groups [11].

A cross-sectional study was conducted on 110 women with diabetes who were randomly divided into two groups with or without sexual dysfunction. Results showed that 53.6% of women had sexual dysfunction and there was a significant relation between albuminuria and sexual dysfunction (*p* = 0.001) and also retinopathy and sexual dysfunction (*p* = 0.007) [12].

A descriptive study among 150 women aged 20–60 years with type 2 diabetes in Iran showed that the mean sexual function score was 22 and 78.7% of women had sexual dysfunction [13]. On the other hand, some studies showed that there is no relation between diabetes type 2 and sexual dysfunction. Ismail et al., in a study on 347 women (174 healthy women and 173 diabetic women), found that prevalence of orgasmic disorders was 13.3% and 10.3% in diabetic and healthy women, respectively, where the differences were not significant [14].

There is not enough information on relation of type 2 diabetes and sexual function and satisfaction in Iran. Therefore, this study was designed to evaluate the relation of type 2 diabetes and sexual function among reproductive age women in Iran.

## 2. Material and Methods

This was a case-control study in which 130 women with type 2 diabetes and 130 healthy women were recruited. The study design was approved by the Ethics Committee of Ahvaz Jundishapur University of Medical Sciences. This study was started in January and completed in June 2014. The inclusion criteria were as follows: married women, healthy women (control group), and women who had type 2 diabetes (case group), body mass index 19.8–29 kg/m^2^, age 15–45, and ability to read and write. Women who were menopausal and had severe mental problems were excluded from the study. The type 2 diabetes diagnosis was confirmed with abnormal fasting blood sugar, abnormal random blood sugar test, and abnormal level of HbA1C. The presence of nephropathy was confirmed by increased urinary albumin excretion in the absence of other kidney diseases [15]. The criteria announced by American Academy of Neurology were considered for detection of neuropathy [16]. The diagnosis of retinopathy was confirmed by criteria recommended by the American Academy of Ophthalmology [17]. All diabetic patients were recruited from diabetic clinic and Research Center affiliated to Ahvaz Jundishapur University of Medical Sciences, Ahvaz, Iran, and had comprehensive medical record, while healthy women were recruited from two public health centers. A written informed consent was obtained before data collection.

Eligible women were requested to complete a demographic questionnaire and female sexual function index (FSFI), while one of the researchers (SY) attended if women had any ambiguity in questions. The FSFI questionnaire has 19 questions for measuring six domains of sexual activity including desire, arousal, lubrication, orgasm, pain, and sexual satisfaction. In this questionnaire, two questions were considered for measuring sexual desire, four for each area of lubrication and arousal, and three questions for each area of orgasm, sexual satisfaction, and pain. A five-point Likert scale was used for scoring. The total score of each domain was multiplied in the certain factor. The factor for desire was 0.6, 0.3 for arousal and lubrication, and 0.4 for other domains. The sexual dysfunction is defined as total number of FSFI < 26 [18]. Fakhri et al. assessed the validity and reliability of this questionnaire in Iran, and results showed that this questionnaire is suitable for measuring sexual function in Iranian women [19]. All participants responded to the Persian version of FSFI questionnaire that was validated in Iran.

## 3. Statistics

All data was entered using SPSS version 16. The chi-square test was used for comparing the nonparametric data, while independent *t*-test was used for comparing parametric figures. The Multivariate Analysis of Covariance (MANCOVA) was used for comparing sexual function between diabetic and healthy women when the components of sexual function were considered as dependent variable and group considered as independent variable. Age, job, education level, economic situation, place of living, and spouse's job have been entered to the MANCOVA model as a covariate. The results were considered significant if *p* < 0.05.

## 4. Results

At the end of study, 106 questionnaires related to the case group and 128 questionnaires related to the control group were completed. Results of this study showed that diabetic patients were significantly older than healthy women (43.3 ± 10.7 versus 37.9 ± 7.7, *p* < 0.001). The number of children did not show any significant difference between two groups (1.91 ± 2.5 versus 2.24 ± 2.4, in the case and control groups, resp., *p* = 0.32). The level of education was significantly higher in the control group compared to the diabetic women (32% of women in the control group had university education compared to 14.2% in the case group, *p* = 0.001). Other demographic characteristics of two groups are listed in Table 1.

Some information about diabetes is presented in Table 2. According to this table, the mean duration of disease was 6.7 ± 5.5 years, 3.8% patients had history of cardiac diseases, and 6.6% of them reported history of renal disease. Forty-one (38.7%) diabetic women were under the insulin therapy, because their disease was not controlled by oral medication. However according to the results of fasting blood sugar (mean: 154.8 ± 74.4) and HbA1C (mean: 9.55 ± 3.4) the disease was not controlled among them. Almost half of the women were suffering from retinopathy (42.2%).

Table 3 indicates information about sexual function between two groups. Results of this study showed that diabetic patients had significantly lower sexual desire, arousal, lubrication, and orgasm and more pain compared to the healthy women (*p* < 0.05). Also diabetic women had lower sexual satisfaction compared to the healthy women (*p* = 0.002). The total score of sexual function was significantly lower in the diabetic women compared to the healthy women (21.25 ± 7.04 versus 22.43 ± 7.6, *p* = 0.004) (Table 3).

## 5. Discussion

This study was designed to explore the relation of type 2 diabetes and sexual function in a case-control study. Results of this study showed that, despite treatment, the diabetes was poorly controlled in the diabetic patients and most women in this group had retinopathy.

Based on the normal distribution assumption in the statistical analyses, results of this study indicated that all areas of sexual function significantly were impaired in diabetic women. Results of other studies also indicated impaired sexual function in diabetic women. A study was conducted by Erol et al. on 72 reproductive age diabetic women and 60 age matched healthy women. Results showed that the most common symptom in diabetic patients was lack of libido (77%) following by vaginal dryness (41.6%) and orgasmic dysfunction (49%) and there was a significant difference in all areas of sexual function between two groups [20]. These results are in line with our results as in our study; all dimensions of sexual function in the diabetic women group were impaired compared to the healthy women.

A study by J. Olarinoye and A. Olarinoye, on 51 women with type 2 diabetes who were compared with 39 healthy women, showed that the total score of sexual function was significantly lower in diabetic patients compared to healthy women (20.5 ± 8.3 compared to 31.2 ± 8.8, *p* < 0.001). Also the scores of all dimensions of sexual function were significantly lower in diabetic patients compared to the healthy women (*p* < 0.05) which shows the interrelationship between various domains of women's sexual functioning [21]. The total score of sexual function in our study was 21.25 ± 7.04 and 22.43 ± 7.6 in the diabetic and healthy women, respectively, which is lower in the healthy women than that in the study of J. Olarinoye and A. Olarinoye. Other studies in Iran also showed that the prevalence of sexual dysfunction among reproductive age women is 27.3% [22]. High blood sugar in diabetic women may lead to reduced tissue and nerve supply especially in sexual organs. This matter affects lubrication and causes reduced sensation in the vagina and therefore painful intercourse and reduced orgasm [23].

A study on 420 women with type 2 diabetes with mean age of 54.4 ± 9.8 years in Tehran, Iran, showed that the average score of FSFI was 14.61 and totally 94.4% of women had sexual dysfunction. Sexual dysfunction was observed in 247 (90.5%) of nonmenopausal women and 153 (98.7%) of postmenopausal women [24]. These results are not in line with our results. The reason for this discrepancy may be due to the fact that the women in the Shadman et al.'s study were older than those in our study. Although authors mentioned that 90.5% of women were not menopausal, according to the mean age of participants they were in perimenopausal age. According to other studies, the sexual function deteriorates in perimenopausal women [25].

A study was conducted by Vafaeimanesh et al., on 110 women with type 2 diabetes in Iran who were divided into two groups of sexual dysfunction and normal sexual function. Results showed that 59 (53.6%) of women had sexual dysfunction. There was no significant difference in the age of women with sexual dysfunction and normal sexual function (48.22 ± 6.6 versus 48.14 ± 5.37 years). There was a significant association between sexual dysfunction and retinopathy (*p* = 0.007), but not with neuropathy [12].

In the present study 41 (38.7%) of patients used insulin for their disease. However the mean of HbA1C was 9.55 ± 3.4 which is much higher than the cut-off (6.5%) which was recommended by American Diabetes Association for diabetes [9]. In the Vafaeimanesh et al.'s study also the mean of HbAIC was 8.20 ± 1.33 which was higher than the cut-off, but lower than what we found in this study. It seems women with type 2 diabetes in Ahvaz need more education about their lifestyle and medications.

## 6. Strength and Limitation of the Study

All women with diabetes type 2 were selected from Diabetes Research Center affiliated to Ahvaz Jundishapur University of Medical Sciences, Ahvaz, Iran, which is a referral center for all diabetic patients in Ahvaz. We did not evaluate the husbands of women regarding chronic diseases. Other studies stated that women with diabetic husband had more sexual dysfunction [26].

## 7. Conclusion

Results of this study showed that the score of all dimensions of sexual function in diabetic patients is lower than that in healthy women. Education and counseling about controlling diabetes and sexual function among diabetic women in reproductive age are recommended.

## Figures and Tables

**Table 1:** The sociodemographic characteristics of participants in two groups of diabetic and healthy women.

Variables	Diabetic *n* = 106	Normal *n* = 128	*p* value
	Mean ± SD or *n* (%)		
Age (y)	43.3 ± 10.7	37.9 ± 7.7	<0.001
Spouse's age (y)	48.6 ± 12.3	45.5 ± 10.2	0.083
Number of children	1.91 ± 2.5	2.24 ± 2.4	0.32
*Job*			
Employee	14 (13.2)	38 (29.7)	0.003
Housewife	92 (86.8)	90 (70.3)	
*Education*			
Primary	25 (23.6)	11 (8.6)	0.001
High school	28 (26.5)	22 (17.2)	
Diploma	38 (35.8)	54 (42.2)	
University	15 (14.2)	41 (32)	
Women who were the only wife of their husband	99 (93.4)	120 (93.6)	0.91
Women whose husbands had another wife	7 (6.6)	8 (6.3)	
*Place of living*			
Rural	9 (8.5)	6 (4.7)	0.23
City	97 (91.5)	122 (95.3)	
*Economic situation*			
Good	40 (37.7)	61 (48)	0.18
Moderate	49 (46.2)	56 (44.1)	
Weak	17 (16)	10 (7.9)	

For the continuous variables the independent *t*-test was used, while, for the categorical data, the chi-square test was used.

**Table 2:** The history of chronic disease, medication, and medical complications among diabetic patients.

Variables	Diabetic *n* = 106 Mean ± SD or *N* (%)
Duration of disease (y)	6.7 ± 5.5
History of cardiac diseases	4 (3.8)
Renal disease	7 (6.6)
*Medication*	
Insulin	41 (38.7)
Metformin + glibenclamide	65 (61.3)
*Laboratories tests*	
Fasting blood sugar	154.8 ± 74.4
2-hour blood sugar	215.6 ± 87
HbA1C	9.55 ± 3.4
*Diabetic complications*	
Nephropathy	9 (8.5)
Retinopathy	45 (42.5)
Heart problem	9 (8.49)
Liver problem	6 (5.6)
Extremities neuropathy	15 (14.1)

**Table 3:** The scores of different dimensions of sexual function in diabetic and healthy women.

Variables	Diabetic *n* = 106	Normal *n* = 128	*p* value
	Mean ± SD		
Sexual desire	3.34 ± 1.37	3.69 ± 1.19	<0.001
Arousal	3.66 ± 1.37	3.79 ± 1.39	<0.001
Lubrication	3.62 ± 1.25	4.05 ± 1.39	0.02
Orgasm	3.68 ± 1.42	3.86 ± 1.47	0.007
Sexual satisfaction	4.01 ± 1.33	4.08 ± 1.36	0.002
Dyspareunia	4.03 ± 1.45	3.99 ± 1.54	0.06
Total score of sexual function	21.25 ± 7.04	22.43 ± 7.6	0.004

For all variables, age, job, education level, economic situation, place of living, and spouse's job have been entered to the MANCOVA model as a covariate.
